# Effects of SGLT2 Inhibitors on Renal Outcomes in Patients With Chronic Kidney Disease: A Meta-Analysis

**DOI:** 10.3389/fmed.2021.728089

**Published:** 2021-11-01

**Authors:** Ning Li, Dan Lv, Xiangjun Zhu, Ping Wei, Yuan Gui, Shijia Liu, Enchao Zhou, Min Zheng, Dong Zhou, Lu Zhang

**Affiliations:** ^1^Division of Nephrology, Affiliated Hospital of Nanjing University of Chinese Medicine, Jiangsu Province Hospital of Chinese Medicine, Nanjing, China; ^2^Division of Nephrology, Department of Medicine, University of Connecticut, School of Medicine, Farmington, CT, United States

**Keywords:** SGLT2 inhibitors, chronic kidney disease, renal outcome, protective effect, meta-analysis

## Abstract

**Introduction:** The effects of sodium-glucose cotransporter-2 (SGLT2) inhibitors on renal outcomes in patients with chronic kidney disease (CKD) were initially demonstrated in recent trials. However, the magnitude of renal benefits for CKD patients with different baseline features and underlying diseases remains unclear.

**Method:** We systematically searched the Embase, PubMed, Web of Science, and Cochrane library databases from inception to April 15, 2021 to identify eligible trials. The primary outcome was a composite of worsening kidney function, end-stage kidney disease (ESKD), or renal death. Efficacy and safety outcomes were stratified by baseline features, such as type 2 diabetes, heart failure, atherosclerotic cardiovascular disease, proteinuria, and renal function.

**Results:** A total of nine studies were included. These studies included 25,749 patients with estimated glomerular filtration rate (eGFR)<60 mL/min/1.73 m^2^ and 12,863 patients with urine albumin-to-creatinine ratio (UACR) >300 mg/g. SGLT2 inhibitors reduced the risk of the primary renal outcome by 30% in patients with eGFR<60 mL/min/1.73 m^2^ (HR 0.70, [95% CI 0.58–0.83], I^2^ = 0.00%) and by 43% in patients with UACR > 300 mg/g (HR 0.57, [95% CI 0.48–0.67], I^2^ = 16.59%). A similar benefit was observed in CKD patients with type 2 diabetes. SGLT2 inhibitors had no clear effects on renal outcomes in patients with eGFR<60 mL/min/1.73 m^2^ combined with atherosclerotic cardiovascular disease (HR 0.74, [95% CI 0.51–1.06], I^2^ = 0.00%). However, they reduced the risk of major renal outcomes by 46% (HR 0.54, [95% CI 0.38–0.76], I^2^ = 0.00%) in patients with atherosclerotic cardiovascular disease and macroalbuminuria (defined as UACR > 300 mg/g). SGLT2 inhibitors did not significantly reduce the risk of major renal outcomes in CKD patients with heart failure (eGFR<60 mL/min/1.73 m^2^: HR 0.81, [95% CI 0.47–1.38], I^2^ = 0.00%; UACR > 300 mg/g: HR 0.66, [95% CI 0.41–1.07], I^2^ = 0.00%). SGLT2 inhibitors showed consistent renal benefits across different levels of eGFR (P interaction = 0.48).

**Conclusion:** SGLT2 inhibitors significantly reduced the risk of the primary outcome in CKD patients. However, for patients with different features and underlying diseases, there exists differences in the renal protective effect.

## Introduction

Chronic kidney disease (CKD) has become a major global public health problem that imposes a heavy burden on families and society. Currently, about 700 million individuals worldwide suffer from CKD, and the incidence will continue to increase (1). Determining how to delay the progression of renal function impairment has become a global focus. Within the past two decades, the only approved renoprotective therapy for CKD patients, notably those with type 2 diabetes, has been renin-angiotensin system (RAS) blockers (2). It is encouraging that in recent years, more and more novel drugs have been developed that provide renoprotection for CKD patients (3–5), including sodium-glucose cotransporter-2 (SGLT2) inhibitors. The emergence of SGLT2 inhibitors has resulted in promising new options for renoprotection.

SGLT2 inhibitors, a new class of glucose-lowering drugs, have been proven to reduce blood glucose, blood pressure, and body mass index (6). Within the past few years, many large-scale trials have been designed to explore cardioprotection and renoprotection in patients with type 2 diabetes or heart failure (7–9). However, most of the primary outcomes of these studies were cardiovascular outcomes. Furthermore, most of the participants did not have CKD. Given these factors, the benefits of SGLT2 inhibitors for renal outcomes in patients with CKD have been questionable.

Over the last 2 years, two large studies (10, 11) that focused on patients with CKD demonstrated the renal benefits of SGLT2 inhibitors in these patients. In the CREDENCE trial (11), the first dedicated trial of an SGLT2 inhibitor in patients with type 2 diabetes and CKD, canagliflozin demonstrated substantial benefits for renal outcomes. In the DAPA-CKD trial (10), data showed that individuals with CKD who received dapagliflozin had a significantly lower risk of a composite of renal outcomes compared with those who received placebo, independent of the presence or absence of type 2 diabetes. However, whether the clinical benefits are related to baseline data, underlying diseases, or renal function remains unknown. It is difficult to draw meaningful conclusions from individual trials. Therefore, we sought to undertake a systematic review to gain more reliable evidence on the renal benefits of SGLT2 inhibitors in CKD patients with different baseline features and underlying diseases.

## Methods

### Study Registration

This systematic review and meta-analysis was designed and guided according to the Preferred Reporting Items for Systematic Reviews and Meta-Analysis (PRISMA) statement (12). Moreover, this meta-analysis was registered in the PROSPERO database (CRD42021247839). No ethical approval or patient consent was required given that all analyses were conducted based on previously published studies.

### Search Strategy

Without language or publication time restrictions, two authors searched for relevant randomized controlled trials that investigated the efficacy of SGLT2 inhibitors in CKD. The following electronic databases were searched: PubMed, Web of Science, Sciencedirect, Embase, and Clinical trials (http://www.clinicaltrials.gov) from their inception to April 15, 2021.

Together with Boolean logical operators, the search was conducted using medical subject headings (MeSH) incorporated with free text terms. The following terms were searched: (“Sodium-Glucose Transporter 2 Inhibitors” OR “sodium glucose transporter ii inhibitor” OR “Sodium–glucose cotransporter 2 inhibitors” OR “SGLT-2 Inhibitors” OR “Inhibitor, SGLT-2” OR “Gliflozins” OR “Canagliflozin” OR “Dapagliflozin” OR “Empagliflozin” OR “luseogliflozin” OR “Ipragliflozin” OR “Tofogliflozin” OR “Sotagliflozin” OR “Remogliflozin” OR “Sergliflozin” OR “Ertugliflozin”) AND “Randomized controlled trial”. Any terms related to “SGLT2i” were searched to prevent leakages.

Meanwhile, we performed several exhaustive searches of major international conference proceedings, grey literature [the non-commercial bibliography of doctors' and masters', technical documents (including government reports)] and clinical trials that may be ongoing or not yet published to minimize loss or omission of suitable articles that met our inclusion criterion. Additionally, the references in each study and meta-analysis of SGLT2 inhibitors were searched for potentially eligible studies. Details on the databases and search strategies are presented in the search strategies supplement. A check was indispensable for the integrity and veracity of studies. All records from the initial search were imported into NoteExpress v3.2.0.7535 to manage and confirm the above information, and was performed concurrently by two independent authors (NL, DL). Discrepancies during this process were resolved through discussion or mediated by a third author (LZ).

### Inclusion Criteria and Literature Selection Process

#### Population

The included population was patients ≥18 years old with CKD, defined as estimated glomerular filtration rate (eGFR) <60 mL/min/1.73 m^2^ or urine albumin-to-creatinine ratio (UACR) > 300 mg/g. There were no race or sex restrictions.

#### Interventions

The included trials required the intervention group to take an SGLT2 inhibitor, and there were no limits on specific doses. Trials of SGLT2 inhibitors in combination with other basic therapeutic agents (such as those for controlling blood pressure or blood sugar) were also permitted.

#### Comparators

Control groups without treatment or treated only with placebos were included. Control groups provided basic treatment were also included.

#### Outcomes

The primary outcomes of this study included: worsening kidney function (defined as doubling of serum creatinine or sustained 40% decline in eGFR), end-stage kidney disease (ESKD) (defined as requirement for chronic dialysis or kidney transplantation, or sustained eGFR below 15 mL/min/1.73 m^2^) or renal death. If the study reported both doubling of serum creatinine and sustained 40% decline in eGFR, we prioritized sustained 40% decline in eGFR as the definition of worsening kidney function. The secondary renal outcome was a composite outcome including worsening kidney function, ESKD, renal death or cardiovascular death, other secondary outcomes including MACE (cardiovascular death, myocardial infarction, and stroke), annualized eGFR slope (annualized difference in eGFR between treatment and control groups), and the percentage of reduction in UACR compared with placebo. The safety outcomes included acute kidney injury, amputation, bone fracture, and volume depletion.

#### Study Design

Trials were restricted to parallel-group multicenter randomized controlled trials. There were no regional or language restrictions. Repetitive studies, case reports, animal experiments, cohort studies, and retrospective studies were excluded.

### Data Extraction and Quality Assessment

We focused on extracting the following information from each study: sample size, age, publication year, study and population features, outcomes of interest, and period of treatment. Data were extracted by three authors (NL, DL, YG) with use of a standardized data form. If we encountered problems during the data extraction process, we consulted two experts in this field (LZ and DZ) for resolution through discussion. For data not available in the original text or appendices, we obtained the relevant secondary analyses by contacting the authors.

The Cochrane quality assessment tool provided by RevMan was used to evaluate the risk of bias in each trial (13). Three authors (NL, DL, SL) independently assessed the risk of bias. The assessment items included random sequence generation, allocation concealment, blinding of participants and personnel, blinding of outcome assessment, incomplete outcome assessment, incomplete outcome data, selective reporting, and other biases. Each item was rated as unknown risk, low risk, or high risk. Analysis of total bias for included studies was also measured. Additionally, the Grading of Recommendations Assessment, Development, and Evaluation (GRADE) framework was used to assess the quality of each outcomes (14). Any discrepancies were adjudicated by a third author (LZ or DZ).

### Data Analysis

If the studies provided corresponding hazard ratio (HR) values, we pooled HRs with 95% confidence intervals (CIs) to evaluate the effect of each trial. If the study only provided the number of events, we used the risk ratio (RR) for the calculation (HR and RR values were analyzed separately and not combined). For continuous variables, weighted mean differences (WMD) were used for analysis. Additionally, we used a random-effects models with application of the DerSimonian–Laird estimator. We assessed heterogeneity between studies using the I^2^ statistics. Values of 25% or less, 25–50%, and 75% and more represented mild, moderate, and high heterogeneity, respectively (15). If the number of included studies was over 10, we conducted a publication bias analysis using the Egger test (16). For different definitions of renal outcomes among the studies, we excluded inconsistent renal outcomes and retained identical renal outcomes for sensitivity analysis. We performed subgroup analyses on primary outcomes to verify if there were any differences between different eGFR subgroups, and whether benefits changed in patients with different underlying diseases [such as type 2 diabetes, heart failure, atherosclerotic cardiovascular disease(ASCVD)]. For each outcome, patients were divided into two groups: UACR > 300 mg/g or eGFR < 60 mL/min/1.73 m^2^. If several studies divided eGFR subgroups into eGFR of 60–45 mL/min/1.73 m^2^ and <45 mL/min/1.73 m^2^, we then combined the HR values of these different eGFR subgroups for analysis. Data were analyzed using STATA version 16.0.

## Results

### Study Selection and Features

**Figure 1:**
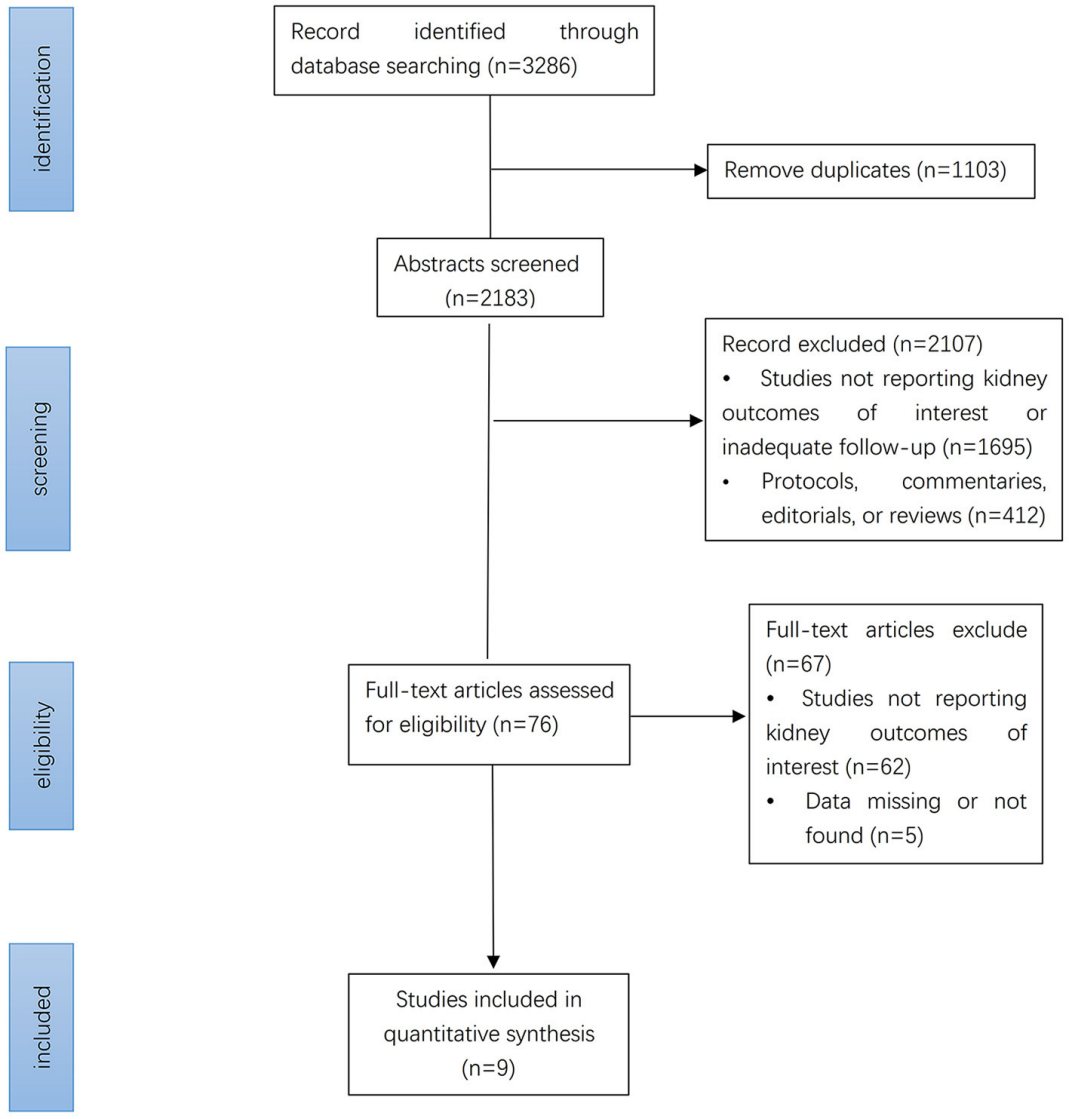
Identification of eligible studies: flow diagram.

A total of 3,286 studies were retrieved by searching the various databases. After screening abstracts and removing duplicates, 76 studies were retrieved. We performed full-text analyses of the studies, and a total of nine were ultimately included according to our strict criteria (Figure 1). Among them, four (8, 9, 17, 18) included patients with type 2 diabetes, two (11, 19) included patients with diabetic kidney disease, two (7, 20) included patients with heart failure, and three (10, 11, 19) included patients with CKD. The detailed screening and retrieval process is shown in the Appendix. The intervention in all studies was SGLT2 inhibitors, and the control groups received matching placebos. All participants were CKD patients. In total, 25,749 had eGFR <60 mL/min/1.73 m^2^ and 12,863 had macroalbuminuria (defined as UACR > 300 mg/g). The lowest eGFR value was 20 mL/min/1.73 m^2^. Mean age among the trials ranged from 61.9 to 69 years. Median follow-up time ranged from 16 to 42 months. Features of the included studies are shown in Table 1.

**Table 1:** Baseline characteristics of patients included in different studies.

**Study**	**Study design**	**Setting**	**Drug dose (mg/day)**	**Median follow up (months)**	**eGFR (ml/min/1.73m^2^)**	**UACR (mg/g)**	**Age (yr)**	**Definition of renal outcomes**
**SGLT2i vs. placebo**								
CANVAS	RCT	Multinational	Canagliflozin 300/100	29	30–59	>300	63.2 ± 8.3/63.4 ± 8.2	≥40% GFR decline, ESKD, renal death
CREDENCE	RCT	Multinational	Canagliflozin 100	31.4	30–59	>300	62.9 ± 9.2/63.2 ± 9.2	Doubling creatinine, ESKD, renal or CV death
DAPA-CKD	RCT	Multinational	Dapagliflozin 10	28.8	25–45	>1000	61.8 ± 12.1/61.9 ± 12.1	≥50% GFR decline, ESKD, renal or CV death
DAPA-HF	RCT	Multinational	Danagliflozin 10	18.2	30–59	–	66.2 ± 11.0/66.5 ± 10.8	≥50% GFR decline, ESKD, renal death
DECLARE–TIMI 58	RCT	Multinational	Danagliflozin 10	50.4	<60	>300	63.9 ± 6.8/64.0 ± 6.8	≥40% GFR decline, ESKD, renal death
EMPA-REG	RCT	Multinational	Empagliflozin 25/10	37.2	30–59	>300	63.1 ± 8.6/63.2 ± 8.8	Macroalbuminuria, doubling creatinine, ESKD, renal death
EMPEROR	RCT	Multinational	Empagliflozin 10	16	20–59	>300	67.2 ± 10.8/66.5 ± 11.2	≥40% GFR decline, ESKD
SCORED	RCT	Multinational	Sotagliflozin 200 OR 400	16.0/15.9	25–59	>300	69	≥50% GFR decline, ESKD, renal death
VERTIS CV	RCT	Multinational	ertugliflozin 15/5	42	30–59	>300	64.4 ± 8.1/64.4 ± 8.0	Doubling creatinine, ESKD, renal death

### Quality Evaluation of Included Studies

There was a certain risk of bias in some of the included studies. Sufficient generation of random sequence was observed in eight trials, while this was unspecified in one trial (19). Adequate blinding of participants and personnel was noted in all studies. Only five trials (8–10, 17, 20) mentioned allocation concealment, while this was unclear in the remaining studies. Relative completeness in the evaluation of outcomes was demonstrated in all studies. The completeness of outcome data in one trial (11) was unclear. Other biases from all of the studies were unclear. Details on overall and individual biases are shown in the Supplementary Figures 1A,B.

### Primary Outcome

**Figure 2:**
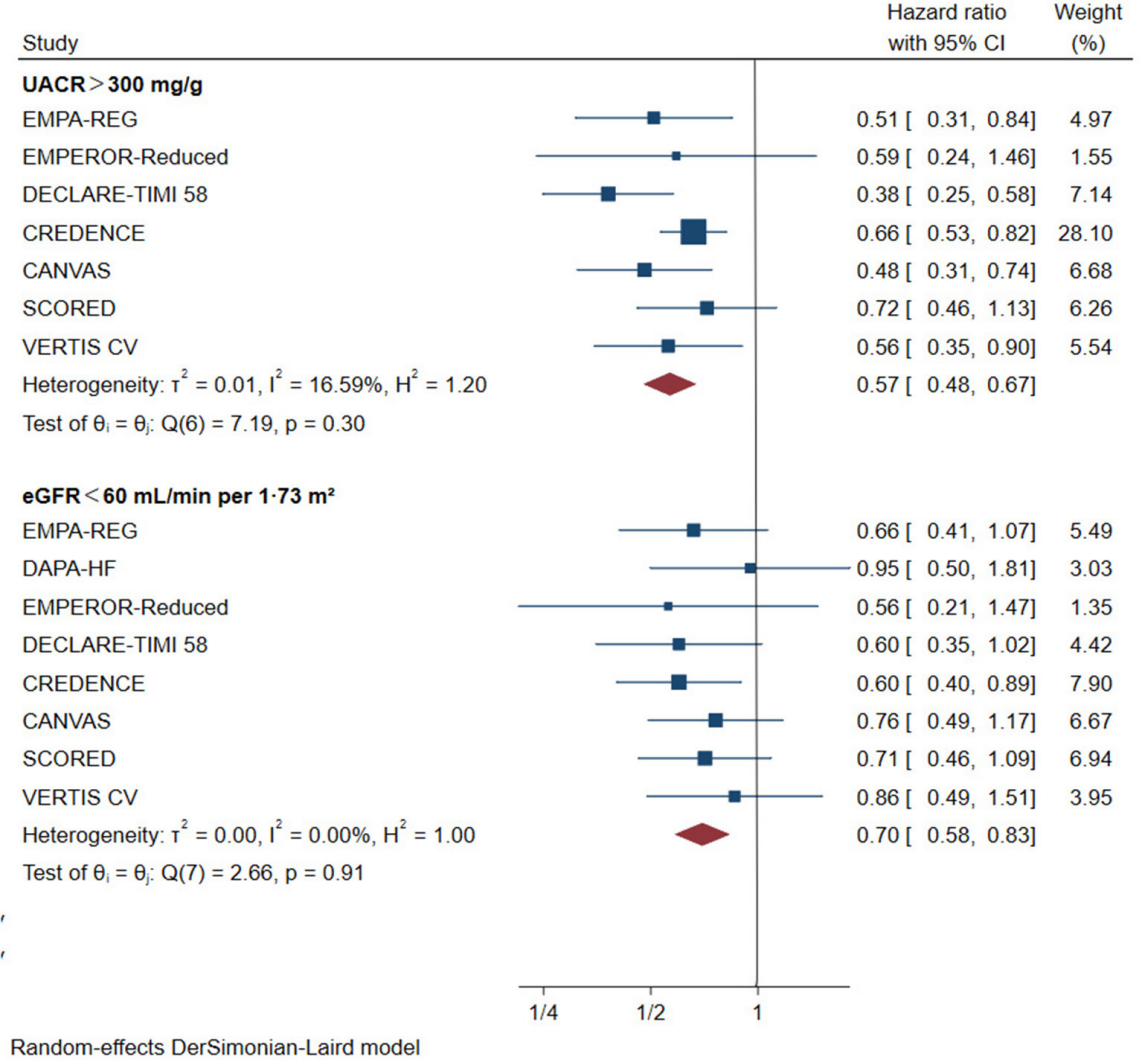
Effect of SGLT2 inhibitors on ESKD, worsening kidney function, or death because of kidney disease. CI, confidence interval; UACR, urinary albumin-to-creatinine ratio; eGFR, estimated glomerular filtration rate. Worsening kidney function: defined as doubling of serum creatinine or sustained 40% decline in eGFR; ESKD, defined as requirement for chronic dialysis or kidney transplantation, or sustained eGFR <15 mL/min/1.73 m^2^.

For patients with eGFR < 60 mL/min/1.73 m^2^, SGLT2 inhibitors reduced the risk of primary renal outcomes by 30% (HR 0.70, [95% CI 0.58–0.83], I^2^ = 0%) compared with placebo (Figure 2). The same benefit (Figure 2) occurred in patients with macroalbuminuria (reduced by 43% compared with placebo, HR 0.57, [95% CI 0.48–0.67], I^2^ = 16.59%). Sensitivity analysis showed that different definitions of worsening kidney function did not alter the risk reduction of primary renal outcomes (Supplementary Table 4).

#### eGFR Subgroups

**Figure 3:**
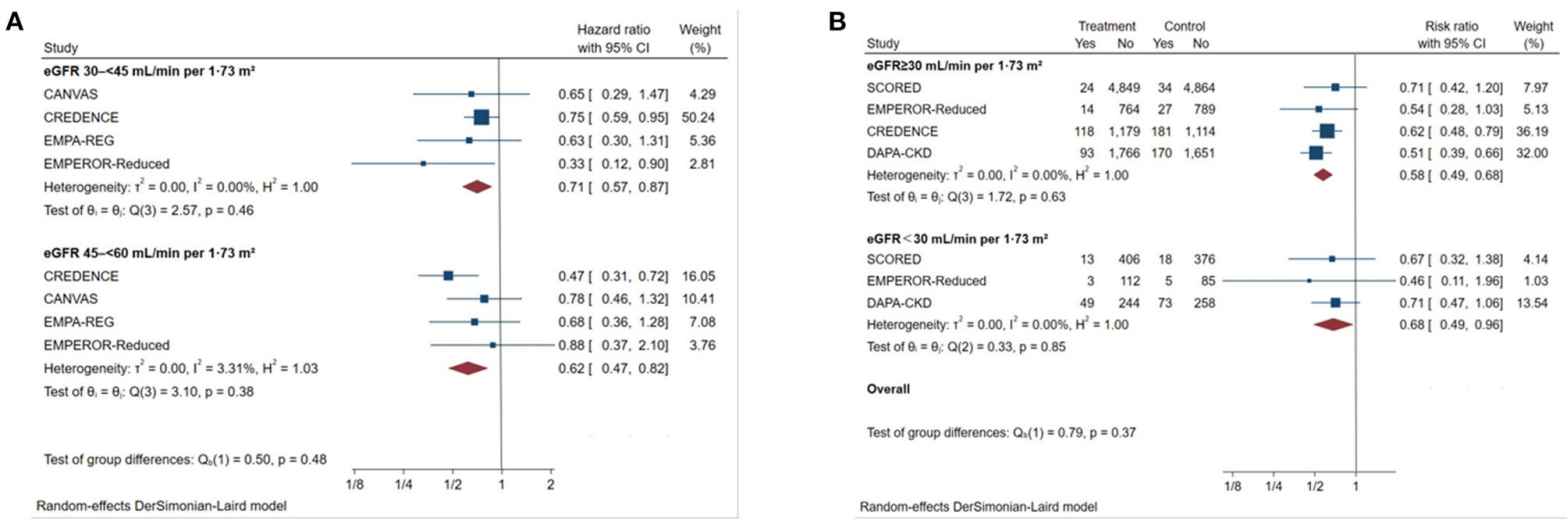
Effect of SGLT2 inhibitors on ESKD, worsening kidney function, or death because of kidney disease across the spectrum of different levels of eGFR. **(A)** Patients with eGFR 45–60 mL/min/1.73 m^2^; **(B)** patients with eGFR <30 mL/min/1.73 m^2^. CI, confidence interval; eGFR, estimated glomerular filtration rate; worsening kidney function: defined as doubling of serum creatinine or sustained 40% decline in eGFR; ESKD, defined as requirement for chronic dialysis or kidney transplantation, or sustained eGFR <15 mL/min/1.73 m^2^.

SGLT2 inhibitors reduced the risk of the primary outcome across different subgroups of eGFR (Figure 3). For patients with eGFR of 45–60 mL/min/1.73 m^2^, the HR was reduced by 38% (HR 0.62, [95% CI 0.47–0.82], I^2^ = 3.31%) and by 29% in patients with eGFR of 30–45 mL/min/1.73 m^2^ (HR 0.71, [95% CI 0.57–0.87], I^2^ = 0%). SGLT2 inhibitors also significantly reduced the risk of primary outcomes among patients with eGFR <30 mL/min/1.73 m^2^ (Figure 3) compared with placebo (RR 0.68, [95% CI 0.49–0.96], I^2^ = 0.00%). The effect of reduction in primary outcomes appeared to be consistent with eGFR ≥30 mL/min/1.73 m^2^ (P interaction = 0.37).

#### Subgroups for Different Underlying Diseases

##### Patients With Type 2 Diabetes

**Figure 4:**
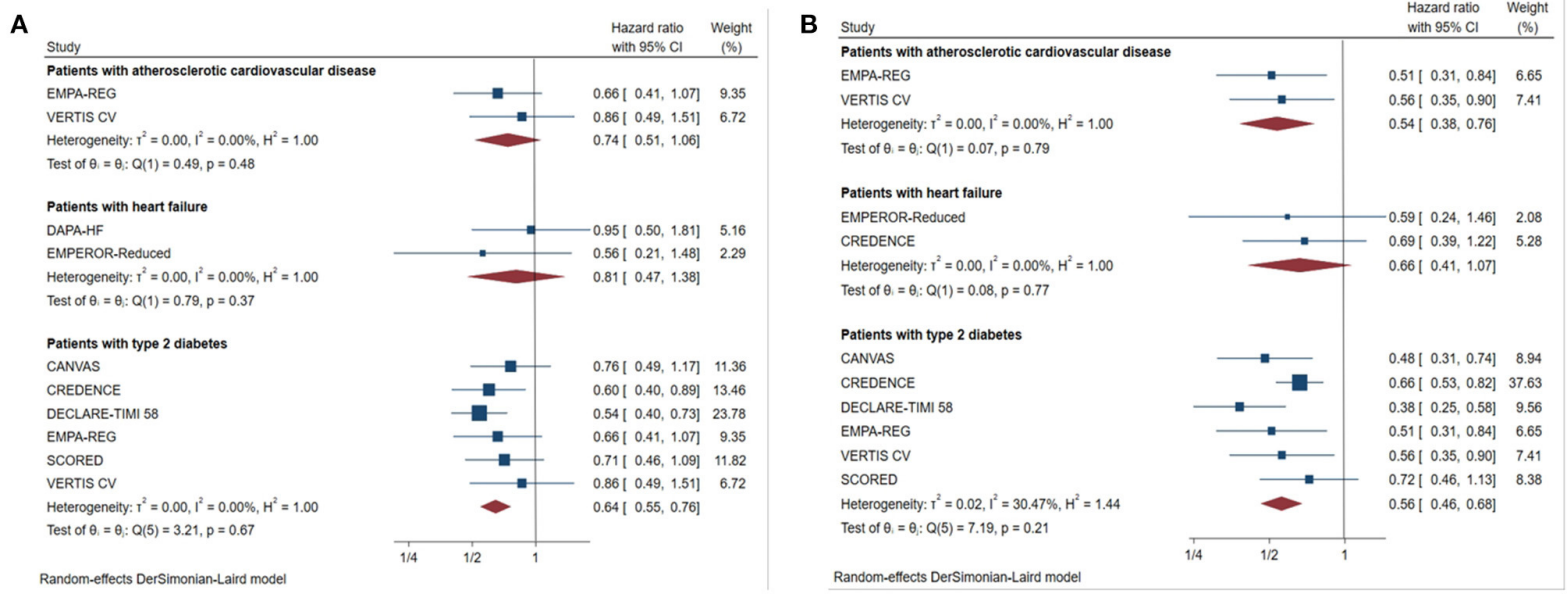
Effect of SGLT2 inhibitors on ESKD, worsening kidney function, or death because of kidney disease in patients with different complications. **(A)** eGFR <60 mL/min/1.73 m^2^; **(B)** UACR > 300 mg/g; CI, confidence interval; worsening kidney function: defined as doubling of serum creatinine or sustained 40% decline in eGFR; ESKD, defined as requirement for chronic dialysis or kidney transplantation, or sustained eGFR <15 mL/min/1.73 m^2^.

For patients with type 2 diabetes, SGLT2 inhibitors reduced the primary outcomes by 36% in those with eGFR < 60 mL/min/1.73 m^2^ (Figure 4, HR 0.64, [95% CI 0.55–0.76], I^2^ = 0.00%) and by 44% in those with UACR > 300 mg/g (Figure 4, HR 0.56, [95% CI 0.46–0.68], I^2^ = 30.47%).

##### Patients With Heart Failure

For patients with heart failure, there was no significant benefit in primary outcome compared with placebo in those with eGFR <60 mL/min/1.73 m^2^ (Figure 4, HR 0.81, [95% CI 0.47–1.38], I^2^ = 0.00%), or UACR > 300 mg/g (Figure 4, HR 0.66, [95% CI 0.41–1.07], I^2^ = 0.00%).

##### Patients With ASCVD

Although the risk of major renal outcomes was reduced by 46% in patients with macroalbuminuria with ASCVD (Figure 4, HR 0.54, [95% CI 0.38–0.76], I^2^ = 0.00%), SGLT2 inhibitors did not significantly reduce the risk in those with eGFR <60 mL/min/1.73 m^2^ combined with ASCVD (Figure 4, HR 0.74, [95% CI 0.51–1.06], I^2^ = 0.00%).

### Secondary Outcomes

SGLT2 inhibitors reduced the risk of the secondary renal outcome (worsening kidney function, ESKD, and renal or cardiovascular death) by 33% (Supplementary Figure 2) in patients with eGFR <60 mL/min/1.73 m^2^ (HR 0.67, [95% CI 0.58–0.78], I^2^ = 0.00%) and by 35% in patients with macroalbuminuria (HR 0.65, [95% CI 0.58–0.73], I^2^ = 0.00%). The HR for MACE was also significantly reduced (Supplementary Figure 2) by 16% in patients with eGFR <60 mL/min/1.73 m^2^ (HR 0.84, [95% CI 0.71–0.99], I^2^ = 54.10%) and 23% in those with UACR > 300 mg/g (HR 0.77, [95% CI 0.67–0.89], I^2^ = 0.00%). The eGFR slope of the SGLT2 inhibitors group appeared to be more stable than that of the control group (Supplementary Figure 2) and this benefit was observed in both those with eGFR <60 mL/min/1.73 m^2^ (WMD 1.67, [95% CI 0.98–2.37], I^2^ = 94.72%) and UACR > 300 mg/g (WMD 3.09, [95% CI 2.10–4.08], I^2^ = 74.88%). However, there was high heterogeneity among the different studies. The percentage of UACR (Supplementary Figure 2) was reduced by 26.92% (WMD 26.92, [95% CI, 7.29–46.55], I^2^ = 78.75%) in patients with eGFR <60 mL/min/1.73 m^2^ compared with placebo and by 31.1% (WMD 31.1, [95% CI, 26.69–35.51], I^2^ = 0.00%) in patients with UACR > 300 mg/g. High heterogeneity was observed in patients with eGFR <60 mL/min/1.73 m^2^ (I^2^ = 78.75%).

### Safety Outcome

According to our results, there were no significant differences in adverse outcomes including amputation, fracture, volume depletion, or acute renal failure between patients with macroalbuminuria receiving SGLT2 inhibitors or placebo (Supplementary Figure 3, acute kidney injury: HR 0.85, [95% CI 0.67–1.08], I^2^ = 0.00%; amputation: HR 1.49, [95% CI 0.72–3.07], I^2^ = 67.61; fracture: HR 0.99, [95% CI 0.74–1.34], I^2^ = 0.00%; volume depletion: HR 1.24, [95% CI 0.98–1.58], I^2^ = 0.00%), or eGFR <60 mL/min/1.73 m^2^ (Supplementary Figure 3, acute kidney injury: HR 0.73 [95% CI 0.47–1.13], I^2^ = 0.00%; amputation: HR 1.10, [95% CI 0.58–2.08], I^2^ = 0.00%; fracture: HR 1.08 [95% CI 0.85–1.38], I^2^ = 0.00%; volume depletion: HR 1.41 [95% CI 0.98–2.02], I^2^ = 0.00%).

### GRADE for the Outcomes

We evaluated all outcome indicators using GRADEpro GDT (https://gradepro.org/). The outcomes of Annualized eGFR slope (Both UACR and eGFR group) and The percentage of reduction in UACR (eGFR group) were low quality, while other outcomes were moderate or high quality (Supplementary Table 5).

## Discussion

Our meta-analysis provides evidence based on current clinical trials for the efficacy and safety of SGLT2 inhibitors on renal outcomes in patients with CKD. For the past 2 decades, only RAS blockers have been shown to exert renoprotective effects in these patients (21, 22). However, the emergence of SGLT2 inhibitors has created new possibilities for patients with CKD. Previously, a meta-analysis (23) included patients with type 2 diabetes with CKD and found that SGLT2 inhibitors significantly reduced the risk of renal outcomes. Our study not only confirmed this result, but also included patients with non-diabetes, which further confirms the efficacy of SGLT2 inhibitors in patients with CKD. We also found that across the spectrum of different eGFR subgroup (eGFR > 30 mL/min/1.73 m^2^), the use of SGLT2 inhibitors was associated with significant renal benefits, and this result is consistent with those from two previous meta-studies (24, 25), which suggests that SGLT2 inhibitors can still provide renal benefits in patients with low eGFR.

Because SGLT2 inhibitors antagonize glucose reabsorption in renal tubules, the action of SGLT2 inhibitors is expected to be eGFR-dependent. For patients with low eGFR, especially those with eGFR <30 mL/min/1.73 m^2^, the use of SGLT2 inhibitors has been controversial. Previously, a *post-hoc* analysis study (26) on canagliflozin showed that in patients with eGFR <30 mL/min/1.73 m^2^, although canagliflozin did not confer an absolute renal benefit compared with placebo, renoprotection was consistent with that in patients with eGFR > 30 mL/min/1.73 m^2^ (P interaction = 0.77). The results from a prespecified analysis of dapagliflozin are similar (27). These observations indicated that patients with eGFR <30 mL/min/1.73 m^2^ may benefit from continued use of SGLT2 inhibitors. A meta-analysis (28) included patients with type 2 diabetes and stage3b-4 CKD found that patients with low eGFR also seen significant renal benefits. To further explore the renal benefits in patients with low eGFR, our study divided the population into stage 3a, 3b and 4, and showed that the protective effect did not change in patients with low eGFR, even in those with stage 4 CKD. These results provide further evidence that use of SGLT2 should be continued in patients with low eGFR population. However, the lower number of participants with eGFR <30 mL/min/1.73 m^2^ and the different underlying diseases may have caused a certain bias.

For CKD patients with different underlying diseases, we found that there were corresponding differences in the magnitude of renal benefits from SGLT2 inhibitors. First, primary renal outcomes were reduced in patients with type 2 diabetes mellitus combined with CKD. This has been confirmed in previous meta-analyses (29). However, our study included additional new large-scale studies and, for the first time, included patients with macroalbuminuria in the analysis. This more strongly confirmed the benefit of SGLT2 inhibitors in this population. Publication of the CREDENCE trial strongly confirmed the renal benefits in patients with type 2 diabetes mellitus combined with CKD. Based on this, 2020 Kidney Disease: Improving Global Outcomes (30) guidelines for treatment of diabetic kidney disease listed SGLT2 inhibitors and RAS blockers as the primary recommendation. Second, our meta-analysis showed that patients with combined heart failure had no significant reduction in primary renal outcome (eGFR <60 mL/min/1.73 m^2^: HR 0.81, [95% CI 0.47–1.38], I^2^ = 0.00%; UACR > 300 mg/g: HR 0.66, [95% CI 0.41–1.07], I^2^ = 0.00%). This may be explained by the following factors: first, heart failure aggravates the progression of CKD. Therefore, the beneficial effects may be attenuated in patients with CKD complicated with heart failure; second, one study (7) included patients with ejection fraction less than 40%, and we believe that lower ejection fraction may interfere with renal outcomes to a certain degree. In addition to patients with heart failure who did not benefit, our study found that there is no significant renal benefit in patients with ASCVD combined with eGFR <60 mL/min/1.73 m^2^ (HR 0.74, [95% CI 0.51–1.06], I^2^ = 0.00%). However, patients with macroalbuminuria were associated with reduced risk of major renal outcomes (HR 0.54, [95% CI 0.38–0.76], I^2^ = 0.00%). Combined with the results in patients with CKD complicated with heart failure, we propose that SGLT2 inhibitors may not provide clinically relevant renal benefits in patients with CKD complicated with CVD, especially those with eGFR <60 mL/min/1.73 m^2^. However, given that the data in this population were primarily from subgroup analysis, and that most of the primary outcomes of these studies were not renal outcomes, the credibility of the results are diminished accordingly.

Regarding renal function, previously, a meta-analysis (31) which included patients with type 2 diabetes and CKD showed that there were no significant changes in eGFR associated with SGLT2 inhibitors compared with placebo. This result is the opposite of ours. We suppose that the reason for the inconsistent results may be due to the risk of bias, and sampling error caused by the small sample size of some studies included in this meta-analysis. In contrast, the studies we included were of higher quality and had a larger sample size. Therefore, the results are of a stronger level of evidence. Currently, the potential mechanism underlying the renoprotective effect is believed to be that the proximal tubule blocks sodium uptake and leads to increased sodium concentration in the distal convoluted tubule, which delivers the sodium signal to the macula densa, leading to afferent arteriolar contraction and decreased glomerular pressure (32). This mechanism is similar to that of RAS blockers, which also exert renoprotective effects by reducing glomerular perfusion pressure (33). Proteinuria is an independent factor for risk of progression of renal disease, and our study confirmed that SGLT2 inhibitors exert a good effect on reducing proteinuria, which may also provide a protective effect for delaying the progression of renal outcomes. In addition, the antihypertensive and anti-inflammatory effects of SGLT2 inhibitors, and their ability to upregulate hypoxic-inducible factor may also have long-term protective effects on the kidney (33, 34). In addition to the renoprotective effect, we found that SGLT2 inhibitors confer favorable cardiovascular benefits in patient with CKD, which significantly reduces the risk of MACE. This suggests that SGLT2 inhibitors can also be used for cardiovascular protection in the CKD population.

Regarding safety outcomes, the results of our study showed that SGLT2 inhibitors did not increase the risk of fracture, amputation, acute kidney injury, or volume depletion. Previously, there were concerns that SGLT2 inhibitors could cause acute kidney injury by regulating hemodynamic mechanisms. Several large studies also demonstrated a significant decrease in eGFR during the early stage of use of SGLT2 inhibitors compared with placebo (8, 9). However, a previous meta-analysis (25) confirmed that SGLT2 inhibitors reduce the risk of acute kidney injury in patients with type 2 diabetes. Another study (35) that focused specifically on acute kidney injury found that use of SGLT2 inhibitors in CKD did not increase the risk of acute kidney injury. Our meta-analysis also showed the same result.

Our meta-analysis had limitations. First, we used combined data rather than individual participant data. Second, there were differences in definitions of endpoints in some studies, which may have had an impact on our results. However, after sensitivity analysis, it was proven there was no substantial impact on our results. Third, the primary outcome of most of the studies was cardiovascular outcomes. In addition, most of the data came from subgroup analyses of major trials, which may reduce the credibility of the results of this study.

## Conclusion

In conclusion, SGLT2 inhibitors significantly reduced the risk of primary renal outcomes in patients with CKD, and this benefit was consistent across the spectrum of different levels of eGFR. Additionally, consistent benefits were observed in patients with type 2 diabetes. However, no significant renal benefit was observed in patients with CKD associated with heart failure. In the population with ASCVD, renal benefits were only observed in CKD patients with macroalbuminuria, whereas no significant benefits were observed in those with eGFR <60 mL/min/1.73 m^2^. In view of the limitations of our study, in the future, additional high-quality studies are needed to confirm the renal benefits of SGLT2 inhibitors in CKD patients with different baseline features and underlying diseases.

## Data Availability Statement

The original contributions presented in the study are included in the article/Supplementary Material, further inquiries can be directed to the corresponding author/s.

## Author Contributions

NL, DL, and LZ contributed to the concept and design of this study. NL, DL, XZ, and PW contributed to the literature search. NL, DL, and YG contributed to the data extraction and risk-of-bias assessment, LZ and DZ acted as consultants for data extraction and literature screening. NL responsible for statistical analysis and writing of the report. MZ assisted in statistical analysis. DL assisted with the writing of the report. EZ reviewed the article and provided critical feedback to shape the report. NL and DL contributed equally to this work and should be considered as co-first authors. All authors read and approved the final manuscript.

## Funding

This study was supported by Special Project of National Clinical Research Base of Traditional Chinese Medicine (No. JDZX2015094).

## Conflict of Interest

The authors declare that the research was conducted in the absence of any commercial or financial relationships that could be construed as a potential conflict of interest.

## Publisher's Note

All claims expressed in this article are solely those of the authors and do not necessarily represent those of their affiliated organizations, or those of the publisher, the editors and the reviewers. Any product that may be evaluated in this article, or claim that may be made by its manufacturer, is not guaranteed or endorsed by the publisher.

## Supplementary Material

Supplementary Figure 1 Risk of bias. Risks of bias in the included studies. **(A)** The authors reviewed the risk of bias for each item in each included study. **(B)** Risks of bias of individual studies. +, low risk of bias; –, high risk of bias; ?, unclear risk of bias.

Supplementary Figure 2 Effect of SGLT2 inhibitors on different secondary outcomes **(A)** Worsening kidney function, ESKD, renal or cardiovascular death; **(B)** Cardiovascular death, myocardial infarction, and stroke; **(C)** Annualized eGFR slope; **(D)** The percentage of reduction in UACR; CI: confidence interval; UACR, urinary albumin-to-creatinine ratio; eGFR, estimated glomerular filtration rate; worsening kidney function: defined as doubling of serum creatinine or sustained 40% decline in eGFR; ESKD, defined as requirement for chronic dialysis or kidney transplantation, or sustained eGFR <15 mL/min/1.73 m^2^.

Supplementary Figure 3 Effect of SGLT2 inhibitors on safety outcomes **(A)** UACR > 300 mg/g; **(B)** eGFR <60 mL/min/1.73 m^2^; CI, confidence interval.

Supplementary Table 1 Kidney outcome ascertainment and adjudication across included studies.

Supplementary Table 2 Definitions for ESKD-based kidney outcomes.

Supplementary Table 3 Difference of slope-based outcomes.

Supplementary Table 4 Sensitivity analyses for the outcome substantial loss of kidney function, ESKD or death due to kidney disease based on different endpoint definitions.

Supplementary Table 5 Grade scores for each outcome.
