# Supplementary material for: Effects of SGLT2 Inhibitors on Renal Outcomes in Patients With Chronic Kidney Disease: A Meta-Analysis
Source: Front Med (Lausanne). 2021 Nov 1;8:728089. doi: 10.3389/fmed.2021.728089 (PMC8591237; doi:10.3389/fmed.2021.728089)
Supplement: Supplementary Figure 1 — Risk of bias. Risks of bias in the included studies. (A) The authors reviewed the risk of bias for each item in each included study. (B) Risks of bias of individual studies. +, low risk of bias; –, high risk of bias; ?, unclear risk of bias. [file Data_Sheet_1.ZIP › ╕╜┬╝/Supplement. Search strategy.docx]

Search strategy

A systematic literature search was then performed by two authors, by searching the following electronic bibliographic databases;

English databases: PubMed, Web of Science, Sciencedirect, Embase and Clinical trials.

**Pubmed**

1# "Sodium-Glucose Transporter 2 Inhibitors"[Mesh]

2# ((((((((((((((((Sodium Glucose Transporter 2 Inhibitors) OR (Sodium-Glucose Transporter 2 Inhibitor)) OR (Sodium Glucose Transporter 2 Inhibitor)) OR (sodium glucose transporter ii inhibitor)) OR (Sodium–glucose cotransporter 2 inhibitors)) OR (Sodium glucose cotransporter 2 inhibitor)) OR (sodium-glucose co-transporter-2 inhibitors)) OR (SGLT-2 Inhibitors)) OR (SGLT 2 Inhibitors)) OR (Gliflozins)) OR (SGLT2 Inhibitors)) OR (Gliflozin)) OR (SGLT-2 Inhibitor)) OR (Inhibitor, SGLT-2)) OR (SGLT 2 Inhibitor)) OR (SGLT2 Inhibitor)) OR (Inhibitor, SGLT2)

3# (((((Tofogliflozin) OR (Apleway)) OR (Deberza)) OR (CSG452)) OR (tofogliflozin hydrate)) OR (tofogliflozin anhydrous)

4# ((((Empagliflozin) OR (Jardiance)) OR (BI 10773)) OR (BI10773)) OR (BI-10773)

5# (((((Dapagliflozin) OR (Farxiga)) OR (Forxiga)) OR (BMS 512148)) OR (BMS512148)) OR (BMS-512148)

6# (((Canagliflozin) OR (Invokana)) OR (Canagliflozin Hemihydrate)) OR (Canagliflozin, Anhydrous)

7# ((Sotagliflozin) OR (LX4211)) OR (LX-4211)

8# ((((luseogliflozin) OR (Lusefi)) OR (TS 071)) OR (TS071 cpd)) OR (TS-071)

9# remogliflozin

10# sergliflozin

11# ((((ertugliflozin) OR (PF-04971729)) OR (Steglatro)) OR (PF 04971729)) OR (PF04971729)

12# (((Ipragliflozin) OR (Suglat)) OR (ASP1941)) OR (ASP-1941)

13# 1# OR 2# OR 3# OR 4# OR 5# OR 6# OR 7# OR 8# OR 9# OR 10# OR 11# OR 12#

((((((("Sodium-Glucose Transporter 2 Inhibitors"[Mesh]) OR (((((((((((((((((Sodium Glucose Transporter 2 Inhibitors) OR (Sodium-Glucose Transporter 2 Inhibitor)) OR (Sodium Glucose Transporter 2 Inhibitor)) OR (sodium glucose transporter ii inhibitor)) OR (Sodium–glucose cotransporter 2 inhibitors)) OR (Sodium glucose cotransporter 2 inhibitor)) OR (sodium-glucose co-transporter-2 inhibitors)) OR (SGLT-2 Inhibitors)) OR (SGLT 2 Inhibitors)) OR (Gliflozins)) OR (SGLT2 Inhibitors)) OR (Gliflozin)) OR (SGLT-2 Inhibitor)) OR (Inhibitor, SGLT-2)) OR (SGLT 2 Inhibitor)) OR (SGLT2 Inhibitor)) OR (Inhibitor, SGLT2))) OR (((((Empagliflozin) OR (Jardiance)) OR (BI 10773)) OR (BI10773)) OR (BI-10773))) OR ((((((Dapagliflozin) OR (Farxiga)) OR (Forxiga)) OR (BMS 512148)) OR (BMS512148)) OR (BMS-512148))) OR ((((Canagliflozin) OR (Invokana)) OR (Canagliflozin Hemihydrate)) OR (Canagliflozin, Anhydrous))) OR (((((luseogliflozin) OR (Lusefi)) OR (TS 071)) OR (TS071 cpd)) OR (TS-071))) OR ((((Ipragliflozin) OR (Suglat)) OR (ASP1941)) OR (ASP-1941))) OR ((((((Tofogliflozin) OR (Apleway)) OR (Deberza)) OR (CSG452)) OR (tofogliflozin hydrate)) OR (tofogliflozin anhydrous))

14# (randomized controlled trial[pt] OR controlled clinical trial[pt] OR randomized[tiab] OR placebo[tiab] OR clinical trials as topic[mesh:noexp] OR randomly[tiab] OR trial[ti]) NOT (animals [mh] NOT (humans [mh] AND animals[mh]))

15# 13# AND 14#

**Embase**

('sodium–glucose cotransporter 2 inhibitor' OR sglt2 OR canagliflozin OR dapagliflozin OR empagliflozin OR luseogliflozin OR ipragliflozin OR tofogliflozin OR Sotagliflozin OR remogliflozin OR sergliflozin OR ertugliflozin) AND ('crossover procedure':de OR 'double-blind procedure':de OR 'randomized controlled trial':de OR 'single-blind procedure':de OR random*:de,ab,ti OR factorial*:de,ab,ti OR crossover*:de,ab,ti OR ((cross NEXT/1 over*):de,ab,ti) OR placebo*:de,ab,ti OR ((doubl* NEAR/1 blind*):de,ab,ti) OR ((singl* NEAR/1 blind*):de,ab,ti) OR assign*:de,ab,ti OR allocat*:de,ab,ti OR volunteer*:de,ab,ti)

**Web of Science**

1# TS=(Sodium-Glucose Transporter 2 Inhibitors OR Sodium Glucose Transporter 2 Inhibitors OR Sodium-Glucose Transporter 2 Inhibitor OR Sodium Glucose Transporter 2 Inhibitor OR Sodium–glucose cotransporter 2 inhibitors OR Sodium glucose cotransporter 2 inhibitor OR sodium-glucose co-transporter-2 inhibitors OR SGLT-2 Inhibitors OR SGLT 2 Inhibitors OR Gliflozins OR SGLT2 Inhibitors OR Gliflozin OR SGLT-2 Inhibitor OR Inhibitor, SGLT-2 OR SGLT 2 Inhibitor OR SGLT2 Inhibitor OR Inhibitor, SGLT2)

2# TS=(Empagliflozin OR Jardiance OR BI 10773 OR BI10773 OR BI-10773)

3# TS=(Dapagliflozin OR Farxiga OR Forxiga OR BMS 512148 OR BMS512148 OR BMS-512148)

4# TS=(Canagliflozin OR Invokana OR Canagliflozin Hemihydrate OR Canagliflozin, Anhydrous)

5# TS=(Tofogliflozin OR Apleway OR Deberza OR CSG452 OR tofogliflozin hydrate OR tofogliflozin anhydrous)

6# TS=(luseogliflozin OR Lusefi OR TS 071 OR TS071 cpd OR TS-071)

7# TS=(Ipragliflozin OR Suglat OR ASP1941 OR ASP-1941)

8# TS=(Sotagliflozin OR LX4211 OR LX-4211)

9# TS=remogliflozin

10# TS=sergliflozin

11# TS=(ertugliflozin OR PF-04971729 OR Steglatro OR PF 04971729 OR PF04971729)

12# 1# OR 2# OR 3# OR 4# OR 5# OR 6# OR 7# OR 8# OR 9# OR 10# OR 11#

13# TS= clinical trial* OR TS=research design OR TS=comparative stud* OR TS=evaluation stud* OR TS=controlled trial* OR TS=follow-up stud* OR TS=prospective stud* OR TS=random* OR TS=placebo* OR TS=(single blind*) OR TS=(double blind*)

14# 12# AND 13#

**Sciencedirect**

(Sodium–glucose cotransporter 2 inhibitor OR SGLT2 OR Canagliflozin OR Dapagliflozin OR Empagliflozin OR luseogliflozin OR Ipragliflozin OR Tofogliflozin OR Sotagliflozin OR remogliflozin OR sergliflozin OR ertugliflozin) AND randomized controlled trial Article types: Research articles

**Clinical trials**

All the clinical trials which may be ongoing or not yet published by searching the term SGLT2i.
