# Supplementary material for: Effects of SGLT2 Inhibitors on Renal Outcomes in Patients With Chronic Kidney Disease: A Meta-Analysis
Source: Front Med (Lausanne). 2021 Nov 1;8:728089. doi: 10.3389/fmed.2021.728089 (PMC8591237; doi:10.3389/fmed.2021.728089)
Supplement: Supplementary Figure 1 — Risk of bias. Risks of bias in the included studies. (A) The authors reviewed the risk of bias for each item in each included study. (B) Risks of bias of individual studies. +, low risk of bias; –, high risk of bias; ?, unclear risk of bias. [file Data_Sheet_1.ZIP › ╕╜┬╝/Table S1. Kidney outcome ascertainment and adjudication across included studies.docx]

Table S1. Kidney outcome ascertainment and adjudication across included studies

ESKD: end-stage kidney disease; eGFR: estimated glomerular filtration rate; RRT: renal replacement therapy

| Study | Pre-specified kidney outcomes | Repeat assessment and confirmation of changes in kidney function and initiation of dialysis | Independent adjudication of kidney outcomes |
| --- | --- | --- | --- |
| CANVAS | • Doubling of serum creatinine, ESKD or death due to kidney disease  • 40% reduction in eGFR, ESKD, or death from renal causes | Yes | Yes |
| CREDENCE | • ESKD, doubling of serum creatinine level, or renal death | Yes | Yes |
| DAPA-CKD | • Decline in estimated GFR of ≥50%, end-stage kidney disease, or death from renal causes | Yes | Yes |
| DAPA-HF | • Worsening renal function (decline in estimated GFR of ≥50%, ESRD, or renal death) | Yes | Yes |
| DECLARE–TIMI 58 | •≥40% decrease in eGFR to <60 ml/min/1.73 m2, ESRD, or death from renal cause | Yes | Yes |
| EMPA-REG | • Doubling of serum creatinine level accompanied by eGFR of ≤45 ml/min/1.73 m2, initiation of renal-replacement therapy, or death from renal disease | No | No |
| EMPEROR | • The rate of the decline in the estimated GFR  • The need for chronic dialysis or renal transplant or a ≥40% decrease in eGFR or a sustained eGFR <15 mL/min/1.73 m2 (if the baseline eGFR was ≥30) or <10 mL/min/1.73 m2 (if the baseline eGFR was <30 mL/min/1.73 m2) | Yes | Yes |
| SCORED | • First occurrence of a sustained decrease of ≥50% in the eGFR from baseline for ≥30 days, long-term dialysis, renal transplantation, or sustained eGFR of <15 ml/min/1.73 m2 for ≥30 days | Yes | Yes |
| VERTIS CV | • Death from renal causes, renal replacement therapy, or doubling of the serum creatinine level | Yes | Yes |
