# Supplementary material for: Effects of SGLT2 Inhibitors on Renal Outcomes in Patients With Chronic Kidney Disease: A Meta-Analysis
Source: Front Med (Lausanne). 2021 Nov 1;8:728089. doi: 10.3389/fmed.2021.728089 (PMC8591237; doi:10.3389/fmed.2021.728089)
Supplement: Supplementary Figure 1 — Risk of bias. Risks of bias in the included studies. (A) The authors reviewed the risk of bias for each item in each included study. (B) Risks of bias of individual studies. +, low risk of bias; –, high risk of bias; ?, unclear risk of bias. [file Data_Sheet_1.ZIP › ╕╜┬╝/Table S2. Definitions for ESKD-based kidney outcomes .docx]

Table S2. Definitions for ESKD-based kidney outcomes

ESKD: end-stage kidney disease; eGFR: estimated glomerular filtration rate; RRT: renal replacement therapy

| Study | Death due to kidney disease | ESKD | Substantial loss of kidney function |
| --- | --- | --- | --- |
| CANVAS | Death with a proximate renal cause | The composite of maintenance dialysis that was sustained for at least 30 days, renal transplantation, or eGFR <15 mL/min per 1·73 m² sustained for at least 30 days | Doubling of serum creatinine/40% decline in eGFR sent for adjudication if sustained for two consecutive measures ≥30 days apart or if occurring on the last available measurement |
| CREDENCE | Death due to kidney disease referred to deaths in patients who reached ESKD who died prior to receiving dialysis or transplantation and no other cause of death was adjudicated | Dialysis for at least 30 days, kidney transplantation, or an estimated GFR of <15 ml per minute per 1.73 m2 sustained for at least 30 days according to central laboratory assessment | Doubling of the serum creatinine level from baseline (average of randomization and prerandomization value) sustained for at least 30 days according to central laboratory assessment |
| DAPA-CKD | Death due to renal disease | Maintenance dialysis for ≥28 days, kidney transplantation, or an estimated GFR of <15 ml per minute per 1.73 m2 confirmed by a second measurement after ≥28 days | A decline of at least 50% in the estimated GFR (confirmed by a second serum creatinine measurement after ≥28 days) |
| DAPA-HF | Death due to renal disease | a sustained [≥28 days] eGFR of <15 ml per minute per 1.73 m2, sustained dialysis, or renal transplantation | A sustained decline in the eGFR of 50% or greater |
| DECLARE–TIMI 58 | Death due to renal disease | Either dialysis for 90 days or more, kidney transplantation, or sustained two measurements at the central laboratory at least 4 weeks apart—eGFR of <15 mL/min per 1·73 m² | Sustained confirmed decrease in eGFR by at least 40% (as confirmed by two tests at the central laboratory at least 4 weeks apart) to less than 60 mL/min per 1·73 m² |
| EMPA-REG | Death due to renal disease | Initiation of RRT (dialysis or transplantation) | A doubling of the serum creatinine level, accompanied by an eGFR of ≤45 ml per minute per 1.73 m2 , as calculated by the MDRD formula |
| EMPEROR | Death due to renal disease | Chronic dialysis or renal transplant or a sustained eGFR <15 mL/min/1.73 m2 (if the baseline eGFR was ≥30) or <10 mL/min/1.73 m2 (if the baseline eGFR was <30 mL/min/1.73 m2 ) | A ≥40% decrease in eGFR |
| SCORED | Death due to renal disease | Long-term dialysis, renal transplantation, or a sustained eGFR of less than 15 ml per minute per 1.73 m2 for at least 30 days | A sustained decrease of at least 50% in the eGFR from baseline for at least 30 days |
| VERTIS CV | Death is due to an identifiable renal etiology | Renal replacement therapy | Doubling of the serum creatinine level |
