# Supplementary material for: Effects of SGLT2 Inhibitors on Renal Outcomes in Patients With Chronic Kidney Disease: A Meta-Analysis
Source: Front Med (Lausanne). 2021 Nov 1;8:728089. doi: 10.3389/fmed.2021.728089 (PMC8591237; doi:10.3389/fmed.2021.728089)
Supplement: Supplementary Figure 1 — Risk of bias. Risks of bias in the included studies. (A) The authors reviewed the risk of bias for each item in each included study. (B) Risks of bias of individual studies. +, low risk of bias; –, high risk of bias; ?, unclear risk of bias. [file Data_Sheet_1.ZIP › ╕╜┬╝/Table S3. Difference of slope-based outcomes.docx]

Table S3. Difference of slope-based outcomes

eGFR: estimated glomerular filtration rate

| Study | Definition | Total slope |
| --- | --- | --- |
| CANVAS | Annual change from Week 6/13 to last available measurement. | - |
| CREDENCE | The on-treatment eGFR slope for the acute and chronic phase was analyzed using a two-slope model with a knot at week 3. | Total slope at week 130 was calculated as a linear contrast of the acute and chronic phases based on the two-slope model. |
| EMPEROR | Chronic slope from week 4 until last eGFR value on treatment. | - |
| EMPA-REG | Chronic slope from week 4  until last eGFR value on  treatment. | - |
