# Supplementary material for: Effects of SGLT2 Inhibitors on Renal Outcomes in Patients With Chronic Kidney Disease: A Meta-Analysis
Source: Front Med (Lausanne). 2021 Nov 1;8:728089. doi: 10.3389/fmed.2021.728089 (PMC8591237; doi:10.3389/fmed.2021.728089)
Supplement: Supplementary Figure 1 — Risk of bias. Risks of bias in the included studies. (A) The authors reviewed the risk of bias for each item in each included study. (B) Risks of bias of individual studies. +, low risk of bias; –, high risk of bias; ?, unclear risk of bias. [file Data_Sheet_1.ZIP › ╕╜┬╝/Table S4. Sensitivity analyses.docx]

Table S4. Sensitivity analyses for the outcome substantial loss of kidney function, ESKD or death due to kidney disease based on different endpoint definitions.

ESKD: end-stage kidney disease; eGFR: estimated glomerular filtration rate

| Renal endpoints | | SGLT2 inhibitors effect on renal endpoints (Hazard ratio (95% CI)) |
| --- | --- | --- |
| Doubling of serum creatinine renal composite | eGFR<60 ml/min per 1.73m2 | 0.66(0.55,0.80) |
|  | UACR>300 mg/g | 0.62(0.52,0.75) |
| 40% eGFR renal composite | eGFR<60 ml/min per 1.73m2 | 0.70(0.56,0.88) |
|  | UACR>300 mg/g | 0.51(0.38,0.69) |
