# Supplementary material for: Effects of SGLT2 Inhibitors on Renal Outcomes in Patients With Chronic Kidney Disease: A Meta-Analysis
Source: Front Med (Lausanne). 2021 Nov 1;8:728089. doi: 10.3389/fmed.2021.728089 (PMC8591237; doi:10.3389/fmed.2021.728089)
Supplement: Supplementary Figure 1 — Risk of bias. Risks of bias in the included studies. (A) The authors reviewed the risk of bias for each item in each included study. (B) Risks of bias of individual studies. +, low risk of bias; –, high risk of bias; ?, unclear risk of bias. [file Data_Sheet_1.ZIP › ╕╜┬╝/Table S5. GRADE Scores for each outcomes.docx]

| **Outcome** | № of participants（study) | Certainty of the evidence (GRADE) | Relative effect (95% CI) |
| --- | --- | --- | --- |
|  |  |  |  |
|  |  |  |  |
| **Worsening kidney function , ESKD or renal death (eGFR group)** | 23831 | ⨁⨁⨁⨁ | **HR 0.70** |
|  | (8 RCTs) | High a | (0.58 to 0.83) |
| **Worsening kidney function , ESKD or renal death (UACR group)** | 11566 | ⨁⨁⨁⨁ | **HR 0.57** |
|  | (7 RCTs) | High a | (0.48 to 0.67) |
| **Worsening kidney function , ESKD, renal or cardiovascular death (eGFR group)** | 8198 | ⨁⨁⨁⨁ | **HR 0.67** |
|  | (4 RCTs) | High a | (0.58 to 0.78) |
| **Worsening kidney function , ESKD, renal or cardiovascular death (UACR group)** | 7249 | ⨁⨁⨁⨁ | **HR 0.65** |
|  | (3 RCTs) | High a | (0.58 to 0.73) |
| **Cardiovascular death, myocardial infarction, and stroke (eGFR group)** | 9522 | ⨁⨁⨁◯ | **HR 0.84** |
|  | (5 RCTs) | Moderate a,b | (0.71 to 0.99) |
| **Cardiovascular death, myocardial infarction, and stroke (UACR group)** | 5930 | ⨁⨁⨁⨁ | **HR 0.77** |
|  | (3 RCTs) | High a | (0.67 to 0.89) |
| **Annualized eGFR slope (eGFR group)** | 8249 | ⨁⨁◯◯ | **MD 1.67** |
|  | (4 RCTs) | Low a,b,c | (0.98 to 2.37) |
| **Annualized eGFR slope (UACR group)** | 6326 | ⨁⨁◯◯ | **MD 3.09** |
|  | (4 RCTs) | Low a,b,c | (2.10 to 4.08) |
| **The percentage of reduction in UACR (eGFR group)** | 4399 | ⨁⨁◯◯ | **MD 26.92** |
|  | (2 RCTs) | Low a,b,c | (7.29 to 46.55) |
| **The percentage of reduction in UACR (UACR group)** | 5186 | ⨁⨁⨁⨁ | **MD 31.10** |
|  | (2 RCTs) | High a | (26.69 to 35.51) |

⨁⨁⨁⨁: high quality; ⨁⨁⨁◯: moderate quality; ⨁⨁◯◯: low quality.

a:due to risk of bias; b: due to inconsistency; c: due to imprecision.

**Table S5. Grade scores for each outcome**
